# *Ganoderma tsugae* Hepatoprotection against Exhaustive Exercise-Induced Liver Injury in Rats

**DOI:** 10.3390/molecules18021741

**Published:** 2013-01-29

**Authors:** Chi-Chang Huang, Wen-Ching Huang, Suh-Ching Yang, Chih-Chi Chan, Wan-Teng Lin

**Affiliations:** 1 Graduate Institute of Sports Science, College of Exercise and Health Sciences, National Taiwan Sport University, Taoyuan 33301, Taiwan; 2 Graduate Institute of Athletics and Coaching Science, College of Sports and Athletics, National Taiwan Sport University, Taoyuan 33301, Taiwan; 3 School of Nutrition and Health Sciences, Taipei Medical University, Taipei 11031, Taiwan; 4 Department of Hospitality Management, College of Agriculture, Tunghai University, Taichung 40704, Taiwan

**Keywords:** *Ganoderma tsugae*, anti-apoptosis, liver protection, exhaustive exercise

## Abstract

Several studies have been shown that accelerated apoptosis is involved in post-exercise lymphocytopenia and tissue damage after high-intensity exercise. *Ganoderma tsugae* (GT) is one of the well-known medicinal mushrooms that possess various pharmacological functions. This mushroom has traditionally been used for health promotion purposes. This study investigates the hepatoprotective effects of GT on exhaustive exercise-induced liver damage. Twenty-four male Sprague-Dawley rats were randomly divided into four groups and designated as exhaustive exercise only (E), exhaustive exercise with low dosage (EL), medium dosage (EM) and high dosage (EH) GT at 0, 0.1875, 0.9375 and 1.875 g/kg/day, respectively. After 30 days all rats were euthanized immediately after an exhaustive running challenge on a motorized treadmill. The rat livers were immediately harvested. Evidence of apoptotic liver cell death was revealed using terminal deoxynucleotidyl transferase dUTP nick end labeling (TUNEL) assay and caspases mediated cascade events. DNA fragmentation, an apoptosis process, can be examined using TUNEL assay. A few TUNEL-positive hepatocytes, compared to the exercise only group, were observed in the livers from exhaustive animals supplemented with GT. Immunoblot analysis also showed that caspase-6-mediated specific cleavage of lamin A/C was increased significantly in the livers of group E, but was significantly decreased in the EM and EH groups. Our observations demonstrate that GT possesses anti-apoptotic and hepatoprotective potential after exhaustive exercise.

## 1. Introduction

Moderate exercise or individual training could be beneficial to heart and lung function, improve athletic performance and reduce the incidence of chronic diseases from the preventive medicine point of view [1]. However, intense or excessive exercise could cause muscle and tissue injury due to excessive free radicals produced from the increase in muscle oxygen consumption during exercise. As early as the 1980s scientists reported that free radical levels in muscle and liver tissue can increase 2- to 3-fold or more following exhaustive exercise [2]. Exhaustive physical exercise induced muscular damage is associated with oxidative stress [3]. The over production of free radicals can decrease endogenous (enzymatic and non-enzymatic) and exogenous antioxidants levels, impair cell function and lead to eventual cell death, resulting in tissue damage or diseased states [4,5]. 

Natural antioxidant research has been notably increased in recent years in search of safe and efficient agents against reactive oxygen species (ROS) over production in biological systems. A previous study reported that antioxidant food could ameliorate the oxidative damage and improve exercise induced muscular damage [6]. The Ganoderma species, including *G. lucidum* and *G. tsugae*, were studied extensively for their anti-tumor [7,8], immunomodulatory [9,10], anti-aginogenesis [11], anti-inflammatory [12] and hepatoprotective effects [13,14]. These two Ganoderma species were also reported to exert anti-oxidative activities in free radical scavenging and reductive capacity [15,16]. However, to our best knowledge, no reports on the effects of *G. tsugae* on exercise-induced biochemical stress *in vivo* are available in the literature. Our data suggests that *G. tsugae* supplementation could increase exercise performance and inhibit lipid peroxidation after exhaustive treadmill exercise. *G. tsugae* can provide anti-apoptotic effects for liver tissue oxidative injuries by means of caspse-6 inactivation, followed by lamin A/C cleavage prevention, and also ameliorate mitochondrial DNA deletion for anti-oxidation. Therefore, we study the ergogenic, anti-oxidative, anti-apoptotic and hepatoprotective properties of *G. tsugae* in a well-established experimental exhaustive exercise challenge model.

## 2. Results 

### 2.1. Body Weight and Daily Intake

Body weight and daily intake data from each experimental group are summarized in Table 1. There were no significant changes in the initial body weight among the E, EL, EM and EH groups. After administration for 30 days, the final body weight of the EM group was significantly higher than group E. We observed that the daily intake was significantly increased in groups EM and EH compared to group E. Therefore, a 30-day supplementation with medium *G. tsuage* dose would increase rat body weight gain. In addition, a 28-day feeding study in rats is a well-established protocol to test the subacute toxicity for drugs or any kind of material. In this study, we performed the different doses of *G. tsugae* supplementation for 30 days not only for providing the efficacy data, but can yield important toxicological data as well. The *G. tsugae* at doses of 0.1875, 0.9375 and 1.875 g/kg daily supplement to SD rats did not caused any death or acute adverse effect on the clinical observation and mortality to the treatment rats. The results suggested that the supplementation with *G. tsuga* treatments should be safe for all test animals.

### 2.2. *G. tsugae* Effect on Exercise Performance in an Exhaustive Treadmill Exercise Test

**Figure 1:**
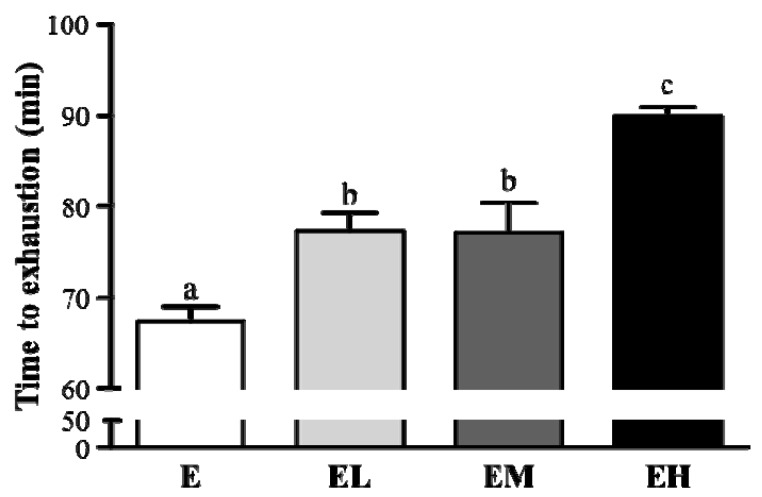
*G. tsugae* supplement effect on the running time to exhaustion. Male SD rats were pre-treated with 0, 0.1875, 0.9375, and 1.875 g/kg of *G. tsugae* (E, EL, EM and EH) for 30 days, and performed an exhaustive treadmill exercise. Data are presented as mean ± SEM of 6 rats in each group. Columns with different letters (a, b) differ significantly, *p* < 0.05 by one-way ANOVA.

Exercise endurance is an important variable in evaluating ergogenic treatment. In our study exercise endurance was studied using an exhaustive treadmill exercise test in rats administered with E, EL, EM and EH were 68.8 ± 2.0, 77.2 ± 2.6, 76.9 ± 4.5 and 89.2 ± 1.3 min, respectively, shown in Figure 1. The exercise time was significantly longer by 1.15-fold (*p* = 0.0039), 1.14-fold (*p* = 0.0046) and 1.33-fold (*p* < 0.0001) with groups EL, EM and EH, respectively, compared to the E group. The maximal running time was increased dose-dependently with the *G. tsugae* doses (*p* < 0.0001).

### 2.3. *G. tsugae* Effect on Clinical Biochemistry Tests after an Exhaustive Treadmill Exercise Challenge

The clinical biochemistry values for group E and mice treated with EL, EM and EH were measured at the experiment end, a single bout of exhaustive treadmill exercise after a 30-day oral feeding trial. As shown in Table 2, no significant differences were exhibited in the E, EL, EM and EH group liver profiles (AST and ALT), cardiac profiles (LDH), muscular functions (CK), and renal profiles (BUN). The results showed that blood glucose and FFA levels in the EM and EL groups, respectively, were significantly lower compared to the E group.

### 2.4. G. tsugae Effects on Oxidative Stress after an Exhaustive Treadmill Exercise Challenge

The various antioxidant enzyme activities such as SOD, CAT, GRD and GPX of erythrocytes, as well as the GSH:GSSG ratio in erythrocyte lysates were not significantly different among the four treatment groups (Table 3). However, the blood TBARS level of the EH group was significantly decreased by 22% compared to the E group. This data showed that *G. tsuage* supplementation could decrease the lipid peroxidation level after an exhaustive exercise challenge.

### 2.5. *G. tsugae* Effect on Hepatic Apoptosis after an Exhaustive Treadmill Exercise Challenge

**Figure 2:**
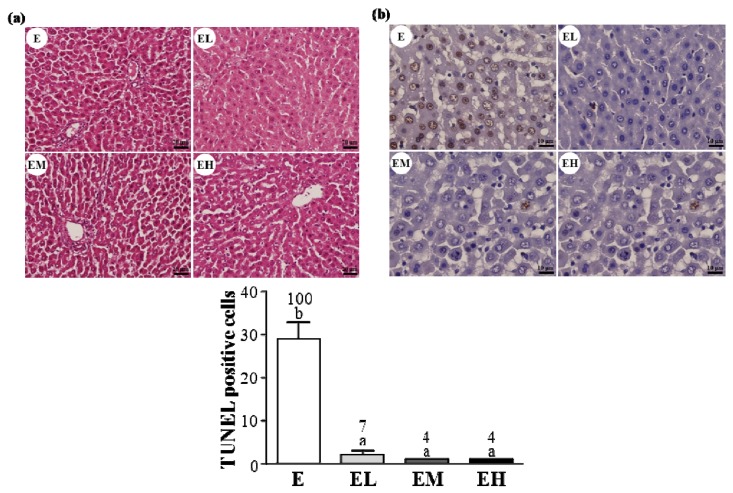
The *G. tsugae* supplement anti-apoptotic effect on liver tissues after exhaustive exercise. (**a**) H&E staining and histology of liver sections from treated rats; (**b**) Liver TUNEL assay after an exhaustive exercise challenge. Male SD rats were pre-treated with 0, 0.1875, 0.9375, and 1.875 g/kg of *G. tsugae* (E, EL, EM and EH) for 30 days and subjected to an exhaustive treadmill exercise. Specimens were photographed using a light microscope (Olympus BX51; Olympus Co., Ltd., Tokyo, Japan). (H&E stain, magnification: ×200, Scale bar, 20 μm; TUNEL stain, magnification: ×400, Scale bar, 10 μm). Results are expressed as a mean of the TUNEL-positive apoptotic cells from 6 rats in each group. Columns with different letters (a, b) differ significantly, *p* < 0.05 by one-way ANOVA.

We examined the liver tissue pathology in each group (Figure 2a) and found no obvious differences among the four treatment groups. However, apoptotic (TUNEL-positive) cells were observed in group E (Figure 2b). Additionally, the number of apoptotic cells was significantly decreased in the EL, EM and EH groups compared to the group E (*p* < 0.0001). In the trend analysis the apoptotic cells were found decreased dose-dependently with the GT doses (*p* < 0.0001). This data showed that the *G. tsuage* supplement could protect against exhaustive exercise induced apoptosis in the liver. 

**Figure 3:**
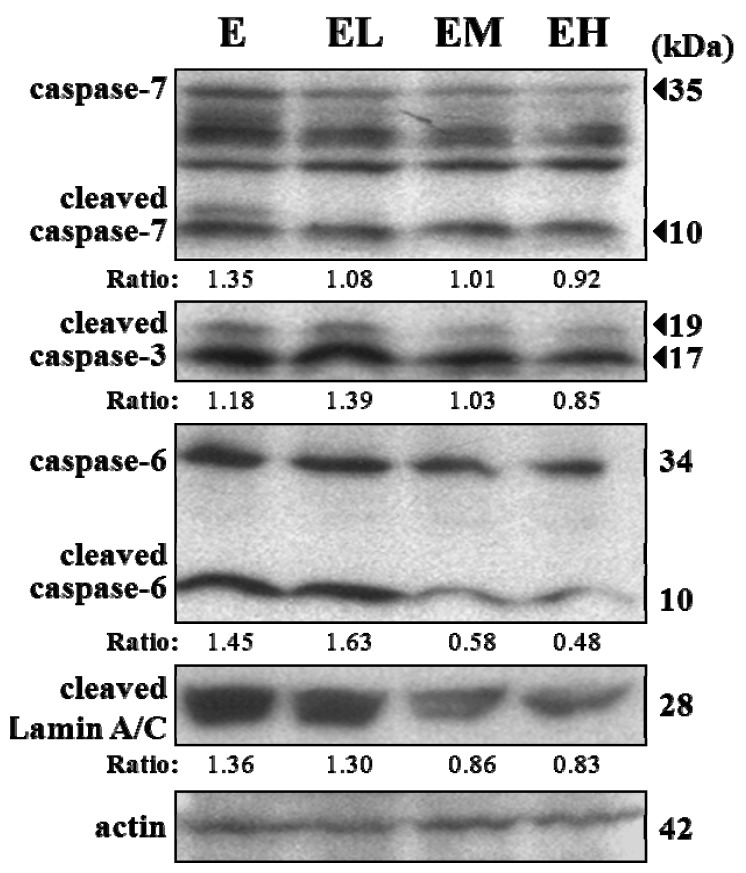
*G. tsugae* supplement effect on caspases-mediated apoptosis pathway in liver tissues after exhaustive exercise. Male SD rats were pre-treated with 0, 0.1875, 0.9375, and 1.875 g/kg of *G. tsugae* (E, EL, EM and EH) for 30 days and subjected to exhaustive treadmill exercise. For western blotting in liver samples, a pooled sample of rat liver proteins (*n* = 6) in each group was loaded on each gel and underwent immunoblotting. The cleaved form of target proteins expression was normalized to that of actin (internal control) by densitometry.

To elucidate the possible mechanisms in exhaustive exercise-induced hepatic apoptosis, we analyzed caspases, the key mediators of apoptosis, protein expression in the livers. The results in Figure 3 showed that the cleaved form of caspases-3 and -6 were significantly decreased in the EM and EH groups compared to group E. The lamin A/C cleavage, activated by caspase-6, was also significantly decreased in the EM and EH groups compared to the E group. The *G. tsuage* treatment greatly inhibited cleaved caspases-3, -6 and lamin A/C level elevation in the EM and EH groups compared to the E group.

### 2.6. G. tsugae Effect on ΔmtDNA^4834^ Deletion of Liver Tissues after an Exhaustive Treadmill Exercise Challenge

**Figure 4:**
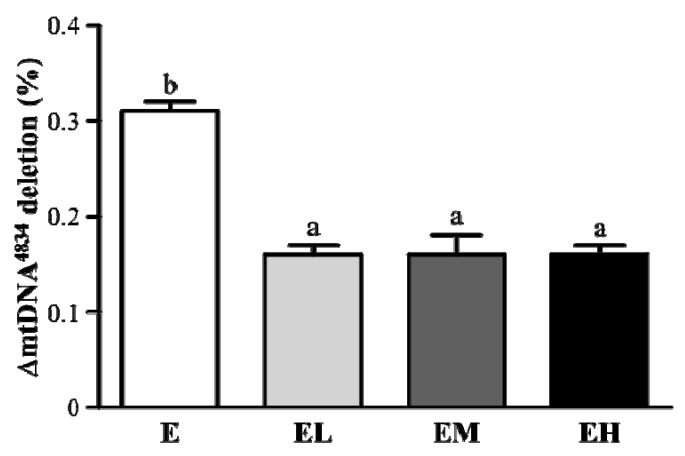
*G. tsugae* effect supplement on ΔmtDNA^4834^ deletion in liver tissues after exhaustive exercise. Male SD rats were pre-treated with 0, 0.1875, 0.9375, and 1.875 g/kg of *G. tsugae* (E, EL, EM and EH) for 30 days and subjected to exhaustive treadmill exercise. Data are presented as mean ± SEM of six rats in each group. Columns with different letters (a, b) differ significantly, *p* < 0.05 using one-way ANOVA.

The free radicals produced by intensive exercise attacked the mitochondrial DNA resulting in specific deletion, such as 4834 bp deletion in rats [4]. As shown in Figure 4 the mitochondrial DNA deletion in the EL, EM and EH groups was significantly decreased by 48.3% compared to group E (*p* < 0.0001). In the trend analysis ΔmtDNA^4834^ deletion in liver tissues was decreased dose-dependently with the GT doses (*p* < 0.0001). The results showed that the *G. tsuage* intervention could protect against exhaustive exercise induced mitochondrial DNA deletion.

## 3. Discussion

The Ganoderma species contain several important bio-active compounds, including polysaccharides, triterpenoids, immunomodulatory peptides, amino acids, alkaloids, and so on [17,18,19]. These compounds present antitumor, immunomodulatory, anti-thrombosis, cholesterol regulation, anti-hyperglycemic and antibacterial activities [20,21,22,23]. Most studies are related to the pharmacological effects of the triterpenoids and polysaccharides. Few studies have focused on ergogenic performance or exercise-induced physiological side effects. A single bout of exhaustive exercise is a well-established protocol to test the maximum physical performance. However, the exhaustive exercise can result in a significant decrease in immune system or increased the oxidative damage, and impair the host homeostasis. This model is a popular and well-known protocol to test the food supplements on anti-fatigue or protective agents for exercise performance. Therefore, we conducted this study to evaluate the effects of *G. tsugae* supplementation on exercise performance and biological functions after a single bout of exhaustive exercise. The biochemical energy metabolism, tissue injury biomarkers, and ergogenic parameter variables were evaluated after exhaustive exercise. The energy metabolic substances including free fatty acid, glucose and tissue glycogen (data not shown) were not significantly increased by *G. tsugae* treatment. There was no significant difference in tissue injury biomarkers and ergogenic parameters. However, *G. tsugae* supplementation significantly elevated exercise performance (Figure 1). The anti-oxidative activity provided by *G. tsugae* could be further discussed to explain this increase in endurance. 

The body’s antioxidant defense system could be improved against exercise-induced oxidative stress and maintain the redox balance state using moderate exercise training [24]. However, this homeostasis system could be interrupted by exhaustive exercise due to the excessive production of ROS in the skeletal muscle [25]. After exhaustive exercise ROS over-accumulation attacks the membrane lipids, resulting in MDA formation which affects normal cellular functions. ROS overproduction has been shown to lead to apoptosis during strenuous exercise [26]. This study found that *G. tsugae* supplementation could effectively inhibit hepatic apoptosis after exhaustive exercise (Figure 2). The apoptosis process could be a defense system used to remove damaged cells, with an over intensive training program triggering mechanism injury, such as inflammatory response, resulting in necrosis or apoptosis. The apoptosis could be partially reversible by therapeutic intervention [27]. A very recent study reported that *G. lucidum* polysaccharide derivatives exhibited antioxidant activity by scavenging free radicals [16]. Our result also demonstrated that *G. tsugae* intervention before exhaustive exercise could significantly ameliorate lipid peroxidation using TBARS assay (Table 3).

This procedure is programed by the serial activation of caspases, a family of cysteine protease, for specific aspartic acid residues. These are two pathways, including death related receptors (Fas ligand, TRAIL receptors, or TNF) and mitochondrial activation, lead to effector caspases 3, 6 and 7 which initiate apoptotic programs [28]. There are also apoptosis associated mediators, such as the lamins, which are nuclear membrane structural components that maintain normal cell functions such as cell cycle control, DNA replication and chromatin organization. The cleavage of lamin A/C activated by caspase-6 results in nuclear deregulation and cell death. Our study found that *G. tsugae* supplementation could substantially suppress the cleavage activation of caspase 3 and 6 and also the proto-form of caspase 6 and 7. The other important indicator, lamin A/C, could be significantly inhibited after intervention. Above all, we believe that the *G. tsugae* supplementation effect that ameliorates apoptosis acts on the downstream apoptotic activation process after exhaustive exercise. 

Mitochondrial DNA (mtDNA) mutations are key players in aging and age related diseases. The human mtDNA 4977 bp deletion (mtDNA 4834 bp deletion in rats) is the most typical mtDNA damage caused by free radicals with a high mutation rate relative to nuclear DNA due to the absence of histones and repair system inefficiencies. In recent studies the oxidative stress induced by oxidants [29], senescence [30], nutritional deficiency [31] and intensive exercise [5] could cause mtDNA deletion *in vitro* and *in vivo*. The protective effect of *G. tsugae* supplementation significantly inhibits mtDNA deletion after exhaustive exercise (Figure 4). 

## 4. Experimental 

### 4.1. Herb Material

*G. tsugae* powder (YK-01) was kindly provided from Yung-Kien Industry Corp, Double Crane Group, Tainan City, Taiwan. The original *G. tsugae* source was selected from a wild growth area in Puli, Nantou, Taiwan and named *G. tsugae* YK-01. According to previous reports, the dry powders from the mycelia and fruit bodies were mixed at a fixed ratio. The *G. tsugae* product was finally encapsulated and preserved at room temperature. One gram of *G. tsugae* product contained approximately 270 mg protein, 0% lipid, 550 mg carbohydrates (including 125 mg polysaccharide), 16.7 mg sodium and 120 mg triterpenoid. A previous study has demonstrated that there are nine triterpenoids isolated from the *G. tsugae* fruiting body and products, and their structures are identified as ganoderic acid A, B, C, D, E, C5, C6, G and ganoderenic acid D by using a reverse phase-HPLC method [18].

### 4.2. Animals, Treatment, and Exhaustive Exercise 

Twenty-four male Sprague-Dawley (SD) rats (five-week old, 230–250 g) were purchased from BioLASCO (A Charles River Licensee Corp., Yi-Lan, Taiwan) and raised in the laboratory animal center of TMU (Taipei Medical University, Taipei, Taiwan) for experiments. All animal experiments conformed to the guidelines of the Institutional Animal Care and Use Committee (IACUC) of TMU. Before the experiments the rats were raised for one week to adapt to the environment and diet. All animals were given distilled water and a standard laboratory diet (No. 5001; PMI Nutrition International, Brentwood, MO, USA) *ad libitum* and appropriately housed in a room maintained at 23 ± 2 °C and humidity 55 ± 10% with a 12-h light-dark cycle.

The 24 male SD rats were randomly divided into four groups (n = 6 per group). All animals were challenged with a single bout of exhaustive treadmill exercise after a 30-day supplementation with a daily oral dose of 0 (E), 0.1875 g/kg (low-dose, EL), 0.9375 g/kg (medium-dose, EM) and 1.875 g/kg (high-dose, EH) of *G. tsugae*, respectively. 

All treatment groups (E, EL, EM, and EH) were practiced to adapt to the treadmill exerciser (T501E, Taiwan) for 15–20 min duration for 10 days before the last exhaustive exercise. The rats in each group were sacrificed for blood and tissues, (liver and muscle), for further analysis. The exercise performance of each rat was measured as running time recorded from the beginning to exhaustion. Electric shock was applied to motivate the rat to run until exhaustion. The rat was considered exhausted when the rat could not run any more after five repeated shock stimulations. The graded exhaustive exercise protocol was adapted according to our previous study [32].

### 4.3. Determination of Blood Biochemical Variables 

The biochemical parameters including plasma lactate, ammonia and glucose levels, and tissue injury indicators including AST, ALT, LDH and creatine kinase (CK). Biochemical activities were analyzed to evaluate the *G. tsugae* supplementation effects on exhaustive exercise. Blood samples were collected from the rat abdominal aortas with indicated treatments after the exhaustive exercise. The plasma was prepared by centrifugation at 1,500 × g, 4°C for 15 min. An auto-analyzer (Hitachi 7060, Hitachi, Tokyo, Japan) was used to analyze and determine the lactate, ammonia and glucose levels, CK activity, AST, ALT, and LDH.

### 4.4. The Glutathione Peroxidase (GPX) Activities

A commercial GPX kit (RS 505; Randox Laboratories, Antrim, UK) was used to measure the antioxidant enzymes activities. The supernatant of various tissue extracts was appropriately diluted, and taken 20 μL diluted sample to mix sequentially with 1 mL pre-mixed reagent (4 mM glutathione, 0.5 U/L glutathione reductase and 0.34 mM NADPH dissolved in 50 mM phosphate buffer, pH 7.2, 4.3 mM EDTA), and 40 μL cumene hydroperoxide. A spectrophotometer was used to (Hitachi U-2000) measure the change in absorption values in 3 min at a wavelength of 340 nm at 37 °C. The GPX activity unit (1U) was defined as the reduction of 1 mol NADPH per minute. The measured values were substituted into the equation and then multiplied using the sample dilution factor. After the tissue supernatant protein was quantified the relative GPX activity of each tissue was presented as U/g unit.

### 4.5. The Glutathione Reductase (GRD) Activities

The antioxidant enzymes GRD activity was measured by commercially available kit (GF2368; Randox Laboratories). The supernatant of various tissue extracts was appropriately diluted and 40 μL diluted sample used to mix sequentially with 1 mL reagent (2.2 mM GSSG), and 200 μL of NADPH (0.17 mM). The spectrophotometer (Hitachi U-2000) measured the change in absorption value in 5 min at a wavelength of 340 nm at 37 °C. The GRD activity unit (1 U) is defined as the reduction of 1 mol of NADPH per minute. The measured values were substituted into the equation and then multiplied using the sample dilution. The GRD relative activity ratio (U/g Hb) was calibrated with hemoglobin.

### 4.6. The Superoxide Dismutase (SOD) Activities

Antioxidant enzymes using commercially available kit (SD 125; Randox Laboratories) measured the plasma SOD activity of indicated treatments after exhaustive exercise. Fifty μL of sample with proper dilution or SOD standard solution was mixed with 1.7 mL substrate solution (50 M xanthine, 25 M I.N.T.) and 250 μL of the enzyme solution (80 U/L, xanthine oxidase). After mixture, the sample was placed in a 1 mL quartz colorimetric tube to record 3 min delta values at 37 °C at a wavelength of 505 nm. The measured values were calibrated into the standard curve, and then multiplied by the sample dilution factor to obtain the relative SOD activities (U/g Hb).

### 4.7. Thiobarbituric Acid Reactive Substances (TBARS) Analysis

The plasma lipid peroxidation status procedure was carried out with a few modifications. One hundred μL of sample was diluted 10-fold with different concentrations of standard (1,1,3,3-tetramethoxypropane, TMP) and placed into a 2 mL micro centrifuge tube. Two hundred μL of 35% TCA and 200 μL Tris-HCl were added sequentially, shaken for 10 min, followed by the addition of 400 μL TBA for 45 min and allowed to react at 95 °C. The sample was taken out and immediately ice-cooled for 5 min at the end of the reaction. The sample was finally added with 400 μL of 20% TCA and centrifuged for 10 min at 3,900 rpm. Two hundred μL of the supernatant was analyzed using an ELISA reader at a wavelength of 530 nm in 96 wells. The measured values were calibrated into the standard curve to obtain the TBARS concentration in the plasma samples.

4.8. mtDNA4834 bp Deletion Determination 

The mtDNA 4834 bp deletion quantity was determined using a TaqMan real-time PCR assay. The total amount of mtDNA in the liver tissue could be used to measure the D-loop region copy number. The D-loop region and the mtDNA 4834 bp deletion were evaluated using the TaqMan PCR assay with specific primers and probes. Each sample was assayed in duplicate and the fluorescence spectrum was continuously monitored. The abundance of mtDNA was calculated on the cycle threshold measurement (CT), and the difference in CT values was used as the relative abundance measurement; △CT (CT_deletion_ − CT_D-loop_) was used to calculate the mtDNA 4834 bp deletion abundance and the mtDNA deletion ratio was calculated as 1/2^△CT^.

### 4.9. Immunoblotting

Apoptosis associated proteins, including caspase-3, caspase-6, caspase-7, and Lamin A/C, were used in Western blotting. All other antibodies including the second antibodies and internal control (β-actin) were from Santa Cruz Biotechnology (Santa Cruz, CA, USA). The protein concentration quantification was conducted using the Bradford method (Bio-Rad Laboratories, Inc., Hercules, CA, USA). The total proteins in each treatment were mixed equally from individuals in each group according to the previous quantification and resolved using 5%–20% gradient SDSPAGE. Samples were then immunoblotted using an enhanced chemiluminescence assay (ECL; Perkin Elmer Life Science., Inc., Boston, MA, USA) and image retrieval using the Gel analysis system (EverGene Biotechnology, Taipei, Taiwan).

### 4.10. TUNEL Assay and H&E Staining of Liver Tissue

The livers were excised and fixed in 10% buffered formalin. Liver tissues were then embedded in paraffin and cut into 4-μm thick slices for the following assay. The serial 4-μm sections were detected using an *In Situ* Cell Death Detection Kit (Roche Diagnostics, Mannheim, Germany) according to the manufacturer’s instructions as described in a previous report [33]. The peroxidase-DAB reactions visualize the apoptotic nuclei and the slides were counterstained with hematoxylin. The same serial liver sections were stained with Hematoxylin and Eosin (H&E) and the results examined using TUNEL and H&E staining using a light microscope equipped with a CCD camera (BX-51, Olympus, Tokyo, Japan).

### 4.11. Statistical Analysis

All data are represented as mean ± SEM. To evaluate the differences among the groups studied. Data were analyzed using one-way ANOVA and the A Cochran-Armitage test for dose effect trend analysis with the Statistical Analysis System (SAS Institute, Cary, NC, USA). *p* < 0.05 was considered statistically significant. 

## 5. Conclusions 

Our data demonstrates that *G. tsugae* could increase the running time to exhaustion in test animals, decrease lipid peroxidation and protect against hepatic apoptosis after exhaustive exercise challenge. These results indicate that *G. tsuga* has liver protective effects and can elevate exercise performance. Although the exact bioactive phytocompounds in *G. tsuga* that contributed to the detailed anti-apoptosis mechanisms and hepatoprotection remain to be elucidated, this study provides science-based evidence to support that *G. tsuga* could be a promising liver protection agent and an ergogenic aid for exercise.

## Figures and Tables

**Table 1:** Initial and final body weight and daily food intake of rats in each group.

Parameters	E	EL	EM	EH
Initial BW (g)	236 ± 5	238 ± 3	243 ± 7	236 ± 4
Final BW (g)	298 ± 12 ^a^	327 ± 15 ^ab^	356 ± 13 ^b^	335 ± 11 ^ab^
Daily intake (g)	18.6 ± 0.7 ^a^	19.9 ± 0.7 ^ab^	20.8 ± 0.6 ^b^	20.9 ± 0.6 ^b^

Data are means ± SEM for n = 6 rats per group. Values in the same line with different superscripts letters (a, b) differ significantly, *p* < 0.05 by one-way ANOVA. All animals were challenged with a single bout of exhaustive treadmill exercise after a 30-day supplementation with a daily dose of 0 (E), 0.1875 g/kg (low-dose, EL), 0.9375 g/kg (medium-dose, EM) and 1.875 g/kg (high-dose, EH) of *G. tsugae*, respectively.

**Table 2:** Biochemical analysis of the *G. tsugae* treatment groups after an exhaustive treadmill exercise challenge.

Parameters	E	EL	EM	EH
AST (U/L)	178 ± 17	159 ± 16	159 ± 21	156 ± 15
AST (U/L)	76.8 ± 10.2	61.3 ± 5.9	59.5 ± 2.3	72.2 ± 15.7
CK (U/L)	162.6 ± 12.9	139.6 ± 44.3	209.5 ± 21.3	161.8 ± 41.3
LDH (U/L)	669 ± 117	682 ± 152	820 ± 114	783 ± 206
Glucose (mg/dL)	53.9 ± 7.4 ^b^	51.3 ± 7.0 ^b^	29.3 ± 1.1 ^a^	36.3 ± 6.2 ^ab^
Lactate (mg/dL)	35.1 ± 5.0	29.6 ± 8.1	41.3 ± 7.0	45.0 ± 7.6
FFA (mM)	0.40 ± 0.04 ^b^	0.20 ± 0.06 ^a^	0.25 ± 0.05 ^ab^	0.39 ± 0.08 ^b^
BUN (mg/dL)	99.4 ± 16.8	67.3 ± 7.6	71.4 ± 8.1	83.6 ± 21.5

Data are means ± SEM for n = 6 rats per group. Values in the same line with different superscripts letters (a, b) differ significantly, *p* < 0.05 by one-way ANOVA. All animals were challenged with a single bout of exhaustive treadmill exercise after a 30-day supplementation with a daily dose of 0 (E), 0.1875 g/kg (low-dose, EL), 0.9375 g/kg (medium-dose, EM) and 1.875 g/kg (high-dose, EH) of *G. tsugae*, respectively.

**Table 3:** *G. tsugae* effect on antioxidant enzymes activities and GSH/GSSG of erythrocytes and plasma TBARS after exhaustive exercise.

Parameters	E	EL	EM	EH
SOD (U/mg Hb)	9.2 ± 2.0	9.1 ± 1.1	10.0 ± 0.9	11.0 ± 2.9
CAT (mU/mg Hb)	47.1 ± 13.7	58.6 ± 18.1	63.7 ± 11.5	40.8 ± 13.2
GRD (mU/mg Hb)	0.51 ± 0.07	0.51 ± 0.16	0.69 ± 0.19	0.55 ± 0.14
GPX (U/g Hb)	230 ± 50	190 ± 37	248 ± 38	228 ± 60
GSH/GSSG	41.2 ± 14.6	59.3 ± 13.9	66.6 ± 14.6	36.9 ± 14.5
TBARS (μM)	4.0 ± 0.4 ^b^	3.8 ± 0.3 ^ab^	3.5 ± 0.2 ^ab^	3.1 ± 0.1 ^a^

Data are means ± SEM for n = 6 rats per group. Values in the same line with different superscripts letters (a, b) differ significantly, *p* < 0.05 by one-way ANOVA. All animals were challenged with a single bout of exhaustive treadmill exercise after a 30-day supplementation with a daily dose of 0 (E), 0.1875 g/kg (low-dose, EL), 0.9375 g/kg (medium-dose, EM) and 1.875 g/kg (high-dose, EH) of *G. tsugae*, respectively.
